# Supplementary material for: Developing a localized resilience assessment framework for historical districts: A case study of Yazd, Iran
Source: PLoS One. 2025 Feb 24;20(2):e0317088. doi: 10.1371/journal.pone.0317088 (PMC11849832; doi:10.1371/journal.pone.0317088)
Supplement: Attachment — Submitted filename: Respons_Reviwers.docx [file pone.0317088.s001.docx]

**Reviewer #1:**

We appreciate the time and effort you dedicated to providing feedback on our manuscript and are grateful for the insightful comments for improvements in our paper. We have incorporated your suggestions. Those changes are visible within the manuscript. Please see below, in green response to the comments and concerns.

1. Please check if these data are correct. Some data in Table 8 are 0, and the Sj is 1.116 of Zartoshtiha in Table 8.

**Response 1:** Thank you for your thorough review. We would like to inform you that the S_j_ value in Table 8 has been corrected, as it was previously a typographical error. Additionally, the data values in Table 8 represent the Q scores for each neighborhood across the seven dimensions, where a Q score of 0 indicates the best possible performance for that neighborhood in the respective dimension (Page 15).

2. Please discuss more about the results and show more contributions of this research and explain how the research results can be used to give values to different area in the world.

We appreciate your comment and understand the concern regarding the contributions of this research results and agree that a more explanation about the research results could provide additional insights. We've updated the manuscript to reflect this (Pages 17-19).

**Reviewer #2:**

We appreciate the time and effort you dedicated to providing feedback on our manuscript and are grateful for the insightful comments for improvements in our paper. We have incorporated your suggestions. Those changes are visible within the manuscript. Please see below, in green response to the comments and concerns.

1. Since this manuscript merged many dimensions and criteria into the framework, please explain how the weights of different parameter determined.

**Response 1:** Thank you for bringing that to our attention. We have incorporated the method for determining the weight of each criterion and dimension into the text, highlighted in Tables 5 and 6 in the manuscript. The manuscript has been updated accordingly to reflect this addition. (Page 13).

2. The study area is quite small. Is this framework proposed by this manuscript suitable for maybe large area? Please explain in detail.

**Response 2:** In response to the esteemed reviewer's comment, the following details can be provided accurately:

- The resilience assessment framework developed in this study is adaptable and scalable. While it has been applied to the historical urban districts of Yazd, the methodologies employed (Fuzzy DEMATEL, Fuzzy ANP, and VIKOR) can be adjusted to accommodate larger areas. The indicators and criteria used in the assessment can be expanded or refined to reflect the diverse conditions and challenges faced in more extensive urban environments.
- The indicators derived from the DROP and BRIC models can serve as a foundation for resilience assessment in larger urban areas. By conducting a comprehensive stakeholder analysis and integrating local context, the indicators can be tailored to capture the specific vulnerabilities and strengths of larger urban districts or regions.
- The framework allows for multi-scale applications. For larger regions, the assessment can be conducted in phases, starting with broader regional evaluations and then focusing on specific districts or neighborhoods. This phased approach enables urban planners and policymakers to prioritize interventions where they are most needed, based on localized resilience scores.
- While applying this framework to larger areas may involve more complex data collection and management, advancements in technology (such as Geographic Information Systems (GIS) and remote sensing) can facilitate the collection of necessary data at a larger scale. These tools can help analyze spatial patterns and identify vulnerabilities across extensive urban landscapes.
- Implementing this framework in larger urban areas can benefit from interdisciplinary collaboration among urban planners, environmental scientists, and social scientists. Such collaboration can enrich the assessment process, ensuring that a wide range of factors influencing resilience is considered and appropriately addressed.
- To further strengthen the applicability of the framework, case studies from larger urban areas could be referenced or conducted. These case studies would validate the framework's effectiveness in different contexts and demonstrate its versatility across various urban environments.

Therefore, while the study initially focused on the historical urban districts of Yazd, the proposed resilience assessment framework is not only suitable for larger areas but also offers a comprehensive and adaptable approach. It emphasizes the need for localized indicators, multi-scale application, and collaborative efforts to enhance urban resilience effectively.

3. For resilience assessment, the following references are suggested to be included.
https://doi.org/10.1016/j.ijdrr.2023.103649
https://doi.org/10.1016/j.uclim.2023.101591
<https://doi.org/10.1016/j.scs.2023.105109>

**Response 3:** We appreciate the comment regarding adding the proposed reference. We added some information in the manuscript. (Page 3).

4. English presentation should be strengthen.

**Response 4:** We carefully revised the text and now the language is with more clarity.
